# Supplementary material for: Complexome profiling of the Chlamydomonas psb28 mutant reveals TEF5 as an early PSII assembly factor
Source: Plant Cell. 2025 Mar 17;37(6):koaf055. doi: 10.1093/plcell/koaf055 (PMC12164590; doi:10.1093/plcell/koaf055)
Supplement: koaf055_Supplementary_Data [file koaf055_supplementary_data.zip › psb28_interactive.zip!/plots/intensity/qualityPlots/peptides/_Cre09.g402775.t1.1.html]

### Cre09.g402775.t1.1

Trivial name:   
SeqCoverage [median]: 20.60  
Mass: 85.09 kDa  
  
 

| Sequence | MissedCleavage | Mass [Da] | wtIntensity | muIntensity | Score |
| --- | --- | --- | --- | --- | --- |
| AASPGLDELQHPLLGEQATLK | 0 | 2187.15 | 0.00 | 1365.89 | 47.75 |
| DSSQELFFR | 0 | 1127.52 | 0.00 | 3072.44 | 83.27 |
| FIHGSATLFPGDVR | 0 | 1515.78 | 0.00 | 4120.29 | 59.19 |
| IGDDFLGLEK | 0 | 1105.57 | 0.00 | 3538.45 | 94.69 |
| LDKKPGAIALR | 2 | 1180.73 | 0.00 | 672.55 | 66.60 |
| LMPDREQIAGFR | 1 | 1431.73 | 0.00 | 577.85 | 46.71 |
| LRGDSSGEKNEDAAQGFVR | 2 | 2034.97 | 0.00 | 852.93 | 38.02 |
| NAGLSAAGGAGAGR | 0 | 1128.56 | 686.66 | 2964.49 | 116.77 |
| TEITRFPHAVLEVK | 1 | 1638.91 | 0.00 | 1843.60 | 78.32 |
| TTSLTVQR | 0 | 904.50 | 674.32 | 8825.72 | 130.77 |
| YVNINYLGFHK | 0 | 1366.70 | 0.00 | 5503.78 | 96.89 |
|  |  |  | 1361.0 | 33338.0 |  |
